# Medicolegal evaluation of pediatric patients with injection neuropathy

**DOI:** 10.55730/1300-0144.6021

**Published:** 2025-04-28

**Authors:** Hızır ASLIYÜKSEK, Caner BEŞKOÇ, Ömer Faruk ŞİMŞEKER, Nihan Hande AKÇAKAYA, Adem KARBUZ

**Affiliations:** 1 Council of Forensic Medicine, Ministry of Justice, İstanbul, Turkiye; 2 Division of Pediatric Infectious Diseases, Department of Child Health and Diseases, Faculty of Medicine, University of Health Sciences, İstanbul, Turkiye

**Keywords:** Injection neuropathy, sciatic nerve injury, malpractice, children, forensic medicine, diclofenac sodium

## Abstract

**Background/aim:**

Injection neuropathy (IN) is a rare but preventable form of iatrogenic nerve injury, particularly affecting the sciatic nerve when intramuscular injections are administered incorrectly. While IN is uncommon in high-income countries, it remains a significant public health issue in Türkiye, especially in pediatric populations. The aim of this study is to evaluate pediatric IN cases referred to the Council of Forensic Medicine and highlight medicolegal aspects to inform clinical and legal preventive strategies.

**Materials and methods:**

We retrospectively analyzed 67 pediatric cases (age range: 1–17 years; mean age: 10.5 years; 57 boys, 10 girls) referred to the Council of Forensic Medicine between January 01/01/2015 and January 01/01/2023, due to suspected injection-related sciatic neuropathy. Demographic data, clinical findings, suspected drugs, injection sites, and medicolegal conclusions were reviewed using descriptive statistics.

**Results:**

Sciatic nerve involvement was confirmed in 64 cases. The most frequently implicated drug was diclofenac sodium (n = 38), followed by sulbactam-ampicillin and clindamycin. In 59 cases, complications were identified; 2 cases were classified as malpractice due to incorrect site or technique. Six cases could not be evaluated due to insufficient medical documentation.

**Conclusion:**

Intramuscular injections, especially in pediatric patients, should not be considered routine procedures. Given the potential for severe complications, oral alternatives should be prioritized. When unavoidable, injections must be performed by qualified professionals considering patient age, muscle mass, and anatomical safety. This study underscores the medicolegal importance of accurate technique and documentation to prevent lifelong disability and litigation.

## 1. Introduction

Injection neuropathy (IN), an iatrogenic injury resulting from intramuscular drug administration, is a preventable yet potentially disabling clinical condition. Among various peripheral nerves, the sciatic nerve is the most frequently affected due to its anatomical proximity to commonly used injection sites, particularly the dorsogluteal region [1,2]. IN is often characterized by acute onset of pain, sensory deficits, and motor impairment in the lower limb, which may progress to permanent neurological deficits and lifelong disability if not managed appropriately

In pediatric populations, IN presents unique challenges. Anatomical and physiological differences, such as reduced muscle mass and variation in fat distribution, make children more vulnerable to injection-related complications. The selection of injection site, needle length, and the experience of the healthcare provider are all critical variables that influence the likelihood of nerve injury [1–8].

Although IN has become increasingly rare in high-income countries due to improved medical training and practice guidelines, it remains a relevant health issue in developing and middle-income countries, including Türkiye. This is attributed in part to the continued reliance on intramuscular drug administration for the treatment of common conditions such as fever and pain, especially in primary care settings [9–14].

Despite being classified as a complication in many cases, injection-related sciatic neuropathy also raises medicolegal concerns. Evaluating these cases in a forensic context allows for a systematic analysis of causative factors and helps distinguish between unavoidable complications and preventable errors.

This study aims to evaluate pediatric cases of suspected injection neuropathy referred to the Council of Forensic Medicine in Türkiye. By examining clinical characteristics, injection practices, and medicolegal assessments, the study seeks to contribute to a better understanding of IN in children and inform both clinical and legal approaches to prevention.

## 2. Materials and methods

This retrospective study included pediatric cases (under 18 years of age) referred to the Council of Forensic Medicine of Türkiye between January 1, 2015, and January 1, 2023, for expert opinion regarding claims of injection-related sciatic nerve injury. The study was approved by the Education and Scientific Research Commission of the Council (Approval No: 21589509/2023/220). Due to the retrospective and anonymized nature of the study, informed consent was waived.

A total of 67 cases were identified in the institutional archives. Each case file was reviewed in detail to extract information regarding age, sex, the clinical indication for injection, the healthcare professional who ordered and administered the injection, the drug used, the injection site, and the presence of postinjection neurological symptoms. Additionally, the expert medical opinions issued by the Council, including the determination of complications or malpractice, were analyzed.

Cases were included if sciatic nerve involvement was confirmed based on clinical records and expert assessment. Cases were excluded if the medical documentation was insufficient to reach a medicolegal conclusion. This resulted in the exclusion of six cases, leaving 61 evaluable cases for analysis.

Descriptive statistics were used to summarize the demographic and clinical features of the cases. No inferential statistical analyses were conducted, as the primary aim was to provide an observational and medicolegal profile of pediatric injection neuropathy cases.

## 3. Results

A total of 67 pediatric cases with suspected injection neuropathy (IN) were reviewed. After excluding 6 cases due to incomplete or missing medical documentation, 61 cases were included in the final analysis. Table 1 summarizes the demographic information of the patients (Table 1). Among these, 57 (85.1%) were boys and 10 (14.9%) were girls, with a mean age of 10.5 years (range: 1–17 years).

The most frequently implicated drug was diclofenac sodium, used in 38 cases (56.7%). Other drugs included sulbactam-ampicillin (n = 12), clindamycin (n = 7), metamizole (n = 4), and various others (e.g., metoclopramide, feniramadol, testosterone, routine vaccines) in single cases (Table 2). Drug information was unavailable in two cases. In terms of injection indication, pain management accounted for 31 cases (46.2%) and upper respiratory tract infections for 30 cases (44.7%).

In 64 cases (95.5%), the injection was administered in the gluteal region; only 2 cases (3.0%) involved the lateral femoral region. One case had no record of injection site. In 60 of the 64 gluteal injections, sciatic nerve involvement was anatomically consistent with dorsogluteal site-related injuries.

Injection orders were given by general practitioners in 40 cases (59.7%) and by pediatricians in 11 cases (16.4%). Regarding administration, 57 injections (85.1%) were performed by nurses and 3 (4.5%) by intern nurses.

In the medicolegal evaluation: 59 cases (88.1%) were classified as complications, including one as a result of patient noncompliance. Two cases (3.0%) were determined to involve malpractice: one due to inappropriate site selection and one due to improper technique. Six cases (9.0%) could not be evaluated due to insufficient medical documentation (Table 3).

## 4. Discussion

Due to the low incidence of intramuscular drug administration, there are not many studies on injection-related sciatic nerve injury from developed countries in recent years. However, in underdeveloped or developing countries, IN is still an important health problem today [9–15]. It has been observed that most of the countries where IN is reported are those that cannot provide adequate conditions for the provision of health services [2,9,10,16–18]. It is believed that iatrogenic nerve injury is common when intramuscular injections are performed under inappropriate conditions by healthcare professionals who lack sufficient knowledge and skills.

Pediatric patients were reported in 370 cases from Spain, 402 from Uganda, 17 from Italy and 33 from Nigeria, in addition to cases under 18 years of age reported in studies without age restrictions [6,11,17,19]. In this study, 67 pediatric cases of IN were evaluated. In the study by Song et al., 80% were febrile illnesses, in the study by Idowu et al., 63.6% were malaria, 12.1% were fever, and in the study by Villarejo and Pascual, infections were common [11,17,19]. In our study, 46.2% of the drugs causing IN were administered for pain, and 44.7% for upper respiratory tract infections.

In the study by Villarejo and Pascual, most of the drugs causing IN were antibiotics and Geyik et al. found that 28.5% of these drugs were nonsteroidal antiinflammatory drugs (NSAIDs), 16.5% were antibiotics, and 14.5% were metamizol [15, 19]. The study by Kline et al. reported that combinations of analgesics and antiemetics, antibiotics and local anesthetics frequently cause IN [1]. Among the drugs most commonly causing IN after their use in Türkiye, nonsteroidal antiinflammatory drugs are the first, followed by analgesics and antipyretics. In this study, 61% of the drugs causing IN were analgesics (56% diclofenac sodium, 7% metamizole), and 28% antibiotics (18% sulbactam-ampicillin, 10% clindamycin). As can be seen, the most commonly used drugs in clinical practice pose the greatest risk.

It was found that the most common cause was drugs containing diclofenac sodium, which is not recommended for use in the pediatric patient group in our study. It was found that diclofenac sodium was administered in 31 cases under the age of 15 years, and 17 of these were administered at the full dose. However, IN developed both in the 17 cases where the full dose was administered and in the 14 cases where lower doses were administered.

In the study by Kakati et al., 50% of injections were given by people without adequate medical qualifications, 18% by doctors and 8% by nurses. In the study by Geyik et al., 83% of the cases where it was known who gave the injection were given by medical staff and 11% by nurses. In the study by Idowu et al., 69.7% of injections were given by nurses, 3% by parents and 1% by doctors. In the study by Pandian et al., injections were self-administered. It was reported that 86% of cases were performed by people without adequate medical qualifications and 14% by doctors and nurses [10–18]. In this study, 85% of injections were administered by nurses and 4% by assistant nurses.

Although there are studies in the literature which show that IN develops after injections given by people who do not have sufficient medical qualifications, there are also studies similar to this study which show that IN can also develop after injections given by people who are highly experienced in this field (doctors, nurses). This point is important because it shows that IN can develop even when injections are given by qualified people. Considering that IN occurs in our country with injections given by healthcare professionals, especially nurses, intramuscular injections should not be considered a routine procedure. It is important that the same care and attention is given to each patient.

In the study by Yalcin et al., which calculated the mean distance of two injection sites to the sciatic nerve in patients with a body mass index (BMI) < 22, the distance was reported to be 18 ± 3.5 cm for the ventrogluteal region and 9 ± 2.3 cm for the dorsogluteal region [20]. In the study by Coskun et al., the distances to the superior gluteal artery and nerve were measured and the dorsogluteal region was found to be closer to the neurovascular structures [21]. As the ventrogluteal region is less commonly used for intramuscular injection applications compared to the dorsogluteal region, there are not many studies in the literature on the advantages and disadvantages of these two regions. It is known that the ventrogluteal region is less preferred than other injection sites [22–24]. In addition, a study of nurses reported that 48% of 593 nurses were not taught the ventrogluteal site during their training [23]. However, there are some differences in the use of the dorsogluteal and ventrogluteal regions, both in terms of drug bioavailability and risk of complications [8]. The reasons for these differences are that peroneal fibres are larger than tibial fibres, contain fewer fascicles and connective tissues, and are located more laterally. For these reasons, peroneal fibres are more involved in dorsogluteal injections.

In our study, 1 case of malpractice was identified due to incorrect site selection and 1 case due to incorrect application technique (malpositioning). However, it is difficult to determine the accuracy of the correct intramuscular injection site, patient position and technique assessment from medical records. Therefore, it should be assessed whether there is any point that supports an error in these matters. In the study, 6 files could not be assessed due to insufficient data. For this reason, it is important to keep and store medical records in our country.

In this study, the injection site was found to be the gluteal region in 64 cases and the laterofemoral region in 2 cases. In one case, information on the injection site was not available. Consistent with the literature, the most commonly used intramuscular injection site was the dorsogluteal region, and the majority of cases (60) involved the sciatic nerve. In these 60 cases, in accordance with anatomical knowledge, the peroneal fibres were predominantly involved in 62.6% of cases, the tibial branch was predominantly involved in 5% of cases and both branches were equally involved in 20% of cases. Even in patients with normal anatomy, this situation, which occurs because the sciatic nerve can be injured when the injection is made in the outer upper quadrant of the dorsogluteal region, is considered by medicolegal experts to be a complication that can occur when injections are carried out even by a suitably equipped and experienced health professional, despite all the care and attention. However, it is considered that the intramuscular use of analgesics, which have no place in pediatric practice and are not recommended for children under the age of 18, cannot be considered a complication even when the correct site is chosen and the appropriate technique is used.

IN, which causes lifelong disability in children, including developmental problems, is also an easily preventable condition. Considering that, according to the World Health Organization, approximately 12 billion injections are administered worldwide each year, 50% of which are unsafe and 75% of which are unnecessary [25], caution should be exercised in the indication of intramuscular treatment. Oral treatment, which is less likely to cause complications in children, should be preferred in the first instance. If intramuscular injections are necessary, they should be administered by experienced healthcare professionals, considering the child’s age and muscle mass, selecting an appropriate site, needle thickness and length, obtaining informed consent and taking the necessary precautions.

Given the permanent damage caused by IN, doctors need to be more careful when choosing the intramuscular route of administration, especially in children. Less harmful drugs should be preferred to drugs such as diclofenac sodium, which is not recommended for use in children under 18 years of age. IN, which can be easily avoided by choosing an alternative injection site other than the dorsogluteal region, will also prevent malpractice lawsuits. To avoid exposure to malpractice lawsuits, which have increased in recent years, the appropriate injection site should be selected and the necessary precautions taken regarding IN, considering the child’s age and muscle mass.

From a medicolegal standpoint, differentiating between complication and malpractice depends not only on the clinical outcome but also on adherence to standard care practices. IN remains a medico-clinical intersection where preventive strategies—both educational and procedural—can significantly reduce the risk of permanent disability and legal disputes.

## 5. Conclusion

Injection neuropathy remains a significant yet preventable cause of permanent disability in children. Although often considered a complication, it may result from suboptimal clinical practices, particularly inappropriate drug selection, injection site choice, and lack of awareness regarding pediatric anatomical differences. This study underscores the importance of adopting safe intramuscular injection techniques, prioritizing oral drug alternatives, and improving professional training for healthcare providers. Accurate documentation and adherence to standard care guidelines are essential not only for patient safety but also to minimize medicolegal disputes. Implementing targeted preventive strategies can substantially reduce the burden of injection-related nerve injuries in pediatric populations.

## Figures and Tables

**Table 1:** Demographic characteristics of the patients.

Parameters	Value
Total cases reviewed	67
Cases included	61
Cases excluded (missing data)	6
Mean age (years)	10.5
Age range (years)	1–17
Sex distribution: Male	57 (93.4%)
Sex distribution: Female	10 (16.4%)

**Table 2:** Drugs implicated in cases.

Drug	Number of Cases	Percentage(%)
Diclofenac sodium	38	56.7%
Sulbactam-ampicillin	12	17.9%
Clindamycin	7	10.4%
Metamizole	4	6.0%
Metoclopramide	1	1.5%
Feniramadol	1	1.5%
Testosterone	1	1.5%
Routine childhood vaccine	1	1.5%
Unknown	2	3.0%

**Table 3:** Medicolegal assessment of the outcomes.

Outcome	Number of Cases	Percentage(%)
Complication (including patient opposition)	59	88.1%
Malpractice (incorrect site selection)	1	1.5%
Malpractice (improper technique)	1	1.5%
Insufficient documentation	6	9.0%

## References

[1] Kline DG Kim D Midha R Harsh C Tiel R Management and results of sciatic nerve injuries: a 24-year experience Journal of Neurosurgery 1998 89 1 13 23 10.3171/jns.1998.89.1.0013 9647167

[2] Tak SR Dar GN Halwai MA Mir MR Post-injection nerve injuries in Kashmir: a menace overlooked Journal of Research in Medical Sciences 2008 13 5 244 247

[3] Yeremeyeva E Kline DG Kim DH Iatrogenic sciatic nerve injuries at buttock and thigh levels: the Louisiana State University experience review Neurosurgery 2009 65 4 A63 66 10.1227/01.neu.0000346265.17661.1e 19927080

[4] Active immunization Kimberlin DW Brady MT Jackson MA Long SS Red Book: 2018 Report of the Committee on Infectious Diseases 31st ed Itasca, IL, USA American Academy of Pediatrics 2018 55 97

[5] Small SP Preventing sciatic nerve injury from intramuscular injections: literature review Journal of Advanced Nursing 2004 47 3 287 296 10.1111/j.1365-2648.2004.03092.x 15238123

[6] Senes FM Campus R Becchetti F Catena N Sciatic nerve injection palsy in the child: early microsurgical treatment and long-term results Microsurgery 2009 29 6 443 448 10.1002/micr.20632 19306387

[7] Pham M Wessig C Brinkhoff J Reiners K Stoll G MR neurography of sciatic nerve injection injury Journal of Neurology 2011 258 1120 1125 10.1007/s00415-010-5895-7 21225276

[8] Jung Kim H Hyun Park S Sciatic nerve injection injury Journal of International Medical Research 2014 42 4 887 897 10.1177/0300060514531924 24920643

[9] Ahuja B Post injection sciatic nerve injury Indian Pediatrics 2003 40 4 368 369 12736415

[10] Pandian JD Bose S Daniel V Singh Y Abraham AP Nerve injuries following intramuscular injections: a clinical and neurophysiological study from Northwest India Journal of the Peripheral Nervous System 2006 11 2 165 171 10.1111/j.1085-9489.2006.00082.x 16787515

[11] Song S Muhumuza MF Penny N Sabatini CS Epidemiology and treatment outcomes in pediatric patients with post-injection paralysis BMC Musculoskelet Disorders 2022 23 1 754 10.1186/s12891-022-05664-4 PMC9354298 35932071

[12] Kretschmer T Heinen CW Antoniadis G Richter HP König RW Iatrogenic nerve injuries Neurosurgery Clinics of North America 2009 20 1 73 90 10.1016/j.nec.2008.07.025 19064181

[13] Hara T Tatebe M Kurahashi T Hirata H Iatrogenic peripheral nerve injuries–Common causes and treatment: A retrospective single-center cohort study Journal of Orthopaedic Science 2021 26 6 1119 1123 10.1016/j.jos.2020.09.009 33115634

[14] Antoniadis G Kretschmer T Pedro MT König RW Heinen CPG Iatrogenic nerve injuries: prevalence, diagnosis and treatment Deutsches Arzteblat International 2014 111 16 273 279 10.3238/arztebl.2014.0273 PMC4010861 24791754

[15] Geyik S Geyik M Yigiter R Kuzudisli S Saglam S Preventing sciatic nerve injury due to intramuscular injection: ten-year single-center experience and literature review Turkish Neurosurgery 2017 27 4 636 640 10.5137/1019-5149.jtn.16956-16.1 27593812

[16] Srinivasan J Ryan MM Escolar DM Darras B Jones HR Pediatric sciatic neuropathies: a 30-year prospective study Neurology 2011 76 11 976 980 10.1212/WNL.0b013e3182104394 21403109 PMC3271574

[17] Idowu AO Ogunrinu AE Akinremi A Aladeyelu OE Kaka B Injection-induced sciatic nerve injury among children managed in a Nigerian physiotherapy clinic: A five-year review African Journal of Physiotherapy Rehabilitation Sciences 2011 3 1 13 16 10.4314/ajprs.v3i1.3

[18] Kakati A Bhat D Devi BI Shukla D Injection nerve palsy Journal of Neurosciences in Rural Practice 2013 4 1 13 18 10.4103/0976-3147.105603 23546341 PMC3579035

[19] Villarejo FJ Pascual AM Injection injury of the sciatic nerve (370 cases) Child’s Nervous System 1993 9 4 229 232 10.1007/BF00303575 8402705

[20] Yalcin E Kose S Akyuz M Comparing the distances of different intramuscular gluteal injection sites to the sciatic nerve Journal of Novel Physiotherapy Physical Rehabilitation 2015 2 1 14 15 10.17352/2455-5487.000019

[21] Coskun H Kilic C Senture C The evaluation of dorsogluteal and ventrogluteal injection sites: a cadaver study Journal of Clinical Nursing 2016 25 7–8 1112 1119 10.1111/jocn.13171 26868292

[22] Engstrom JL Giglio NN Takacs SM Ellis MC Cherwenka DI Procedures used to prepare and administer intramuscular injections: a study of infertility nurses Journal of Obstetric Gynecologic Neonatal and Nursing 2000 29 2 159 168 10.1111/j.1552-6909.2000.tb02036.x 10750682

[23] Beecroft PC Redick SA Intramuscular injection practices of pediatric nurses: site selection Nurse Educator 1990 15 4 23 28 2377326

[24] Roldán-Chicano MT Rodríguez-Tello J Cebrián-López R Moore JR Del Mar García-López M Adverse effects of dorsogluteal intramuscular injection versus ventrogluteal intramuscular injection: A systematic review and meta-analysis Nursing Open 2023 10 9 5975 5988 10.1002/nop2.1902 37452553 PMC10415997

[25] Miller MA Pisani E The cost of unsafe injections Bulletin of the World Health Organization 1999 77 10 808 811 10593028 PMC2557745

